# Systematic review-based guideline “Parkinson’s disease” of the German Society of Neurology: diagnostic use of transcranial sonography

**DOI:** 10.1007/s00415-024-12502-1

**Published:** 2024-07-04

**Authors:** Uwe Walter, Kai F. Loewenbrück, Richard Dodel, Alexander Storch, Claudia Trenkwalder, Günter Höglinger, for the German Parkinson Guideline Group

**Affiliations:** 1 Department of Neurology, Rostock University Medical Center, Gehlsheimer Str. 20, 18147 Rostock, Germany; 2 Deutsches Zentrum für Neurodegenerative Erkrankungen (DZNE) Rostock/Greifswald, Rostock, Germany; 3 Center for Transdisciplinary Neurosciences Rostock (CTNR), Rostock, Germany; 4 Faculty of Medicine Carl Gustav Carus, Department of Neurology, University Hospital, Technische Universität Dresden, Dresden, Germany; 5 Service de Neurologie, Centre Hospitalier de Luxembourg, Luxembourg, Grand Duchy of Luxembourg; 6 Chair of Geriatric Medicine and Center for Translational Neuro- and Behavioral Sciences, University Duisburg-Essen, Essen, Germany; 7 Paracelsus-Elena-Klinik, Kassel, Germany; 8 Department of Neurosurgery, University Medical Center Göttingen, Göttingen, Germany; 9 Department of Neurology, LMU University Hospital, Ludwig-Maximilians-Universität (LMU) München, Munich, Germany; 10 Deutsches Zentrum für Neurodegenerative Erkrankungen (DZNE), Munich, Germany; 11 Munich Cluster for Systems Neurology (SyNergy), Munich, Germany

**Keywords:** Essential tremor, Neuroimaging, Parkinson’s disease, Parkinsonism, Systematic review, Transcranial ultrasound

## Abstract

**Background and objective:**

Transcranial brain parenchyma sonography (TCS) has been recommended as a tool for the early and differential diagnosis of Parkinson’s disease (PD) in German and European clinical guidelines. Still, the brain structures to be examined for the diagnostic questions and the requirements for being a qualified investigator were not specified in detail. These issues have now been addressed in the 2023 update of the clinical guideline on PD by the German Society of Neurology (DGN).

**Methods:**

The recommendations were based on a systematic literature review following PRISMA (Preferred Reporting Items for Systematic Reviews and Meta-Analyses) guidelines.

**Results:**

Three diagnostic questions were defined: (1) What is the accuracy of TCS in the differential diagnosis of PD versus atypical and secondary Parkinsonian syndromes? (2) What is the accuracy of TCS in the differential diagnosis of PD versus essential tremor? (3) What is the accuracy of TCS in the diagnosis of PD in persons with typical early symptoms, compared with the diagnosis established by clinical follow-up? The brain structures to be assessed and the level of recommendation were formulated for these questions. The training requirements for being regarded as qualified TCS investigator were stipulated by the responsible medical societies (German Society of Ultrasound in Medicine, DEGUM; German Society for Clinical Neurophysiology and Functional Imaging, DGKN). Finally, the recommendations for these diagnostic questions reached strong consensus (each ≥ 97%) of the guideline committee. Here, the details of review and recommendations are presented.

**Conclusion:**

The updated guideline clarifies the diagnostic uses and limitations of TCS in PD.

**Supplementary Information:**

The online version contains supplementary material available at 10.1007/s00415-024-12502-1.

## Introduction

**Fig. 1:**
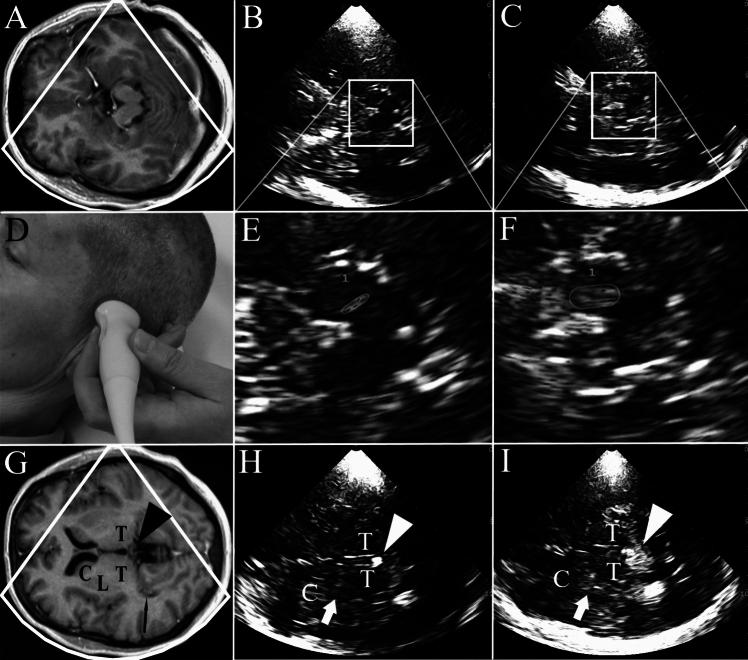
Transcranial sonography (TCS) in the diagnosis of Parkinson’s disease (PD). **A** MRI of midbrain axial transection corresponding to the TCS images shown in (**B**) and (**C**). **B** Axial TCS scan at midbrain level in an individual with normal aspect of substantia nigra (small echogenic area, SN–). This finding is typical for essential tremor and multiple system atrophy. **C** Axial TCS scan at midbrain level in an individual with enlarged echogenic size of substantia nigra (hyperechogenicity, SN +). This finding is typical for PD. **D** Position of the ultrasound transducer for diagnostic TCS in PD. **E** Zoomed TCS image of midbrain shown in (**B**). The echoic area of substantia nigra is traced for automated measurement. **F** Zoomed TCS image of midbrain shown in (**C**). The echoic area of substantia nigra is traced for automated measurement. **G** MRI of basal-ganglia axial transection corresponding to the TCS images shown in (**H**) and (**I**). *C* caudate nucleus, *L* lenticular nucleus, *T* thalamus, arrow head: pineal gland. **H** Axial TCS scan at basal-ganglia level in an individual with normal aspect of lenticular nucleus (weakly echogenic, LN−; arrow). This finding is typical for PD. **I** Axial TCS scan at basal-ganglia level in an individual with increased echogenicity of lenticular nucleus (LN + ; arrow). This finding is frequent in atypical Parkinsonian syndromes

Since the first description of the characteristic transcranial sonography (TCS) finding of enlarged echogenic appearance (“hyperechogenicity”) of the substantia nigra in Parkinson’s disease (PD) [1], numerous studies have underpinned the diagnostic value of TCS in PD [2–14]. Specific advantages of TCS compared to other brain imaging methods are its non-invasiveness and low interference with patients head movements. Applying high-end ultrasound systems with standardized settings [9–14], high image resolution of echogenic deep brain structures of up to 0.7 × 1.1 mm is achieved [15]. For planimetric measurement of substantia nigra echogenicity by an experienced investigator high intra-rater (ICC 0.97 and 0.93, respectively, for both hemispheres) and inter-rater reliability (ICC 0.84 and 0.89) have been demonstrated [16]. For the diagnostic work-up of Parkinsonian syndromes, two standardized transtemporal axial scanning planes are used: the mesencephalic plane in which the substantia nigra is assessed, and the third ventricular/thalamic plane in which the ventricular system and the basal ganglia are assessed (Fig. 1). The diagnostic evaluation of these structures by TCS has been described earlier in detail [9–14]. Meanwhile, TCS of substantia nigra, basal ganglia and ventricles is well established as a supportive diagnostic tool in PD. TCS by an experienced investigator has been included in the European clinical guidelines as an optionally recommended method for:

(i) the differential diagnosis of PD from atypical and secondary Parkinsonian syndromes,
(ii) the early diagnosis of PD in clinically unclear cases with Parkinsonian motor signs, and
(iii) the detection of subjects at risk for PD,

including asymptomatic mutation carriers for monogenic forms of PD, with preferably combining TCS with other screening procedures for (iii) [17]. In the clinical guideline of the German Society of Neurology (DGN), TCS was listed already in 2008 as a facultative diagnostic method for supporting the diagnosis of PD at early motor disease stages [18]. The subsequent German guideline 2012 listed TCS as a facultative diagnostic method for (i) the discrimination of PD from atypical Parkinsonian syndromes and (ii) supporting the diagnosis of PD at early motor stages [19]. In the updated German guideline 2016, TCS was recommended as an optional method for the premotor, early and differential diagnosis of PD, based on systematic review of studies published until 2010 [20]. The guideline required an experienced TCS investigator, and proposed a first criterion for this, i.e. an investigator having performed TCS on 100 individuals with or without PD [20]. Still, these guidelines did not exactly define the diagnostic questions and respective brain structures to be assessed on TCS, nor the requirements in education and training for being regarded as qualified TCS investigator [17–20]. These needs have now been addressed with the recently issued 2023 update of the German clinical guideline (https://register.awmf.org/de/leitlinien/detail/030-010) [21]. In the present article, the specified diagnostic questions for TCS, the results of systematic literature reviews and the new guideline recommendations are reported.

## Methods

### Definition of the diagnostic questions

The guideline coordinating committee initially set one diagnostic question for TCS:

1. What is the accuracy of TCS in the diagnosis of PD compared with the diagnosis established by long-term clinical follow-up?
  The drafting authors of the chapter “Brain parenchyma sonography” (UW, KL) were requested in December 2020 to confirm or revise the diagnostic question. Considering the previous guidelines [17–20], and the scientific evidence available in early 2021, the following more detailed diagnostic questions were proposed by the chapter authors and confirmed by the coordinating committee in May 2021:
2. What is the accuracy of TCS in the differential diagnosis of PD versus atypical and secondary Parkinsonian syndromes?
3. What is the accuracy of TCS in the differential diagnosis of PD versus essential tremor?
4. What is the accuracy of TCS in the diagnosis of PD in persons with typical early symptoms*, compared with the diagnosis established by clinical follow-up? (*early motor signs of PD, hyposmia, depression, REM sleep behavior disorder)

The key questions were converted into the “PICOS” format for further search (see below). For each of these diagnostic questions, a systematic review was conducted in accordance to the Preferred Reporting Items for Systematic Reviews and Meta-Analyses (PRISMA) guidelines for diagnostic test accuracy [22, 23]. These systematic reviews were not registered.

### Data sources and search strategy

Systematic literature searches were performed through PubMed to identify studies eligible for inclusion. All records published until 31.12.2021 were included in the initial search. Search terms are outlined in Table S1 in the supplementary materials (Online Resource 1). No restrictions on language, or study type were specified on the search protocol. The following PICOS criteria were used as a framework to formulate the literature search strategies to ensure comprehensive searches:

- P (Population): adults (>18y) with (suspected) PD and/or (if applicable) atypical/secondary Parkinsonian disorders and/or (if applicable) essential tremor.
- I (Intervention): transcranial B-mode sonography (TCS) of brain parenchyma.
- C (Comparison): clinical diagnosis of PD established by movement disorder specialists based on international consensus criteria (established/confirmed at follow-up visits).
- O (Outcomes): detection/discrimination of PD on TCS.
- S (Studies): original articles (including observational studies, randomized control trials), systematic reviews, meta-analyses, and case series.
- The title and abstract of the detected records were screened for relevance and full text articles were retrieved for those that passed the inclusion criteria. For articles from the same group containing a search period overlap and similar data sets, only the most recent article was included to avoid duplication of data. Narrative reviews, editorials, short communications, case studies, and articles for which full text was not retrievable were excluded. The studies identified on the initial search were entered in an Excel sheet (Microsoft, Redmond, Washington, USA).

### Study analysis and guideline establishment

The studies listed in the Excel sheet were then further assessed for their relevance and reported data by the chapter authors, who in the first step independently evaluated the reports. A backward citation and a forward citation (dating until 30th July 2023) were used when appropriate to include pertinent articles. Subsequently, consensus was achieved by the chapter authors on which of the identified studies were regarded as relevant for the systematic review. The variables for which data were sought were: the number of participants per diagnostic group undergoing TCS, participant characteristics (age, gender, disease duration), the ultrasound system applied for TCS, the qualification of investigator, degree of blinding of investigators to diagnosis, the definition (cut-off criterion on planimetry) of substantia nigra hyperechogenicity, the diagnostic gold standard, the duration of follow-up, and the number of cases who met the diagnostic endpoint(s). The data analysis of each finally approved study included the assessment of sensitivity, specificity, positive and negative predictive values (PPV, NPV), as well as positive and negative likelihood ratios (LR + , LR – ) with respect to the referring diagnostic question. Strengths and weaknesses of each study were commented. These data and comments were listed in a table, separately for each diagnostic question. Based on this, a concise report on the levels of evidence of the relevant studies was compiled, and the resulting recommendations were formulated. The chapter tables and reports were then included in the draft of the complete guideline and send out for reading to all guideline chapter lead authors (n = 34) and delegates of involved medical societies (n = 20), who formed the consensus voting committee. In 3 web-based consensus meetings all guideline chapters had to be presented and defeated by the referring lead authors, and the strength level of each recommendation and, if applicable, the required further editions of the referring chapter were consented by committee. Considering all requested revisions, a final voting was performed in the committee web-conference, and the degree (percentage) of consensus was documented.

## Results

### Identification of relevant studies

For the diagnostic question 1, the PubMed search identified 11 records, out of which three studies were included after screening, and another 11 studies were included from citation search. Details are given in Figure S1A in the supplementary materials (Online Resource 2). For the diagnostic question 2, the PubMed search identified five records, out of which none were included after screening, however, another five studies were included from citation search (Figure S1B, Online Resource 2). For the diagnostic question 3, the PubMed search identified 30 records, out of which two studies were included after screening, and another three from citation search (Figure S1**C**, Online Resource 2).

### Guideline report and recommendations

1. *What is the accuracy of TCS in the differential diagnosis of PD versus atypical and secondary Parkinsonian syndromes?*
  Background: Particularly in the early motor disease stages the clinical discrimination of PD from atypical and secondary Parkinsonian syndromes may be difficult. Therefore, there is a strong need of additional diagnostic tools to increase diagnostic certainty.
  Evidence basis: In this literature search, one large longitudinal cohort study, one systematic review with meta-analysis, nine cross-sectional studies, two small longitudinal cohort studies and two reviews, but no studies with histologically proven diagnosis were identified.
  Result: TCS supports the diagnostic discrimination of PD versus atypical and secondary Parkinsonian syndromes in the first years of (motor) disease since the TCS findings are specific already in this disease stage [10]. The reliability of TCS in this application is dependent on the qualification of the investigator [16, 24]. Therefore, TCS is to be performed by a qualified investigator. If TCS is performed only of substantia nigra, PD (typically hyperechoic substantia nigra, SN +) can be discriminated best from multiple system atrophy (MSA; typically normal substantia nigra, SN-) [3, 25]. If also other atypical Parkinson syndromes are considered (progressive supranuclear palsy, PSP, corticobasal degeneration, CBD), TCS only of substantia nigra is less specific since in PSP and CBD SN + is more frequent [25, 26]. A meta-analysis of 71 studies on more than 5,000 patients yielded only a sensitivity of 75% and a specificity of 70% of substantia nigra TCS in the discrimination of PD from atypical Parkinsonian syndromes [27]. However, several longitudinal and cross-sectional studies have demonstrated that the additional TCS assessment of lenticular nucleus and third-ventricle width increases the diagnostic accuracy (Table 1) [10, 28–33]. The combined finding of hyperechoic lenticular nucleus (LN +) with at least one of two other (SN- or third-ventricle width > 10 mm) discriminates multiple system atrophy and progressive supranuclear palsy best from PD [29]. For the discrimination of PD and dementia with Lewy bodies TCS of substantia nigra is helpful, TCS of basal ganglia or ventricles does not add diagnostic value [32, 34]. The characteristic TCS finding in dementia with Lewy bodies is a bilateral-symmetric SN + (asymmetry index < 1.15, individual ratio of the larger echogenic size divided by the lesser echogenic size); the diagnostic specificity, however, is limited. In patients with vascular Parkinsonism typically bilateral SN- is found [4, 35, 36]. It has been discussed that patients with suspected vascular Parkinsonism who, however, exhibit SN + on TCS might have PD [36]; this remains to be studied systematically. In general, TCS does not completely discriminate PD from other Parkinsonian syndromes, therefore the individual clinical course and other diagnostic findings need to be considered.

**Table 1:** Studies on TCS for the differential diagnosis of PD versus atypical and secondary Parkinsonian syndromes

Study	Diagnostic groups	Diagnostic gold standard	TCS criterion	Sensitivity, specificity, PPV; LR + ; LR-	Quality of study, comment
*PD versus atypical Parkinsonian syndromes (MSA, PSP, CBD)*					
[3]	PD (n = 25) vs. MSA/PSP (n = 23)	Clinical diagnose by an independent movement disorder specialist	SN + (moderate or marked)	96%, 91%, 92%; 11.0; 0.04	Low (small patient number, included patients with possible PSP)
[28]	MSA/PSP (n = 40) vs. PD (n = 88)	Clinical diagnose by an independent movement disorder specialist	Combined finding of LN + and SN-	70%, 99%, 97%; 61.6; 0.30	Moderate (retrospective design, included patients with possible MSA and PSP)
[29]	MSA/PSP (n = 40) vs. PD (n = 134)	Clinical diagnose by an independent movement disorder specialist	SN-	72%, 98%, 91%; 32.4; 0.28	Moderate (no follow-up, included patients with possible MSA and PSP)
	MSA/PSP (n = 39) vs. PD (n = 125)	Do	Combined finding of LN + and SN-	59%, 100%, 100%	Do
	MSA/PSP (n = 39) vs. PD (n = 125)	Do	Combined finding of LN + and SN- or LN + and 3 V +	82%, 98%, 94%; 51.3; 0.18	Do
[25]	PD (n = 43) vs. MSA/PSP/CBD (n = 13)	Clinical diagnose at 12-months follow-up	SN + (moderate or marked)	91%, 85%, 95%; 5,89; 0,11	Moderate (small number of patients with atypical PS)
[10]	PD (n = 353) vs. MSA/PSP (n = 147) (pooled data of five studies)	Clinical diagnose by an independent movement disorder specialist	SN + (moderate or marked)	92%, 80%, 91%; 4.50; 0.10	Moderate (no or only short follow-up, variable degree of blinding of raters)
	MSA/PSP (n = 79) vs. PD (n = 213) (pooled data of two studies)	Do	Combined finding of LN + and SN-	65%, 99%, 98%; 137.5; 0.36	Do
[30]	MSA/PSP/CBD (n = 17) vs. PD (n = 13)	Clinical diagnose at 9-months follow-up	LN +	82%, 85%, 87%; 5.35; 0.21	Low (small patient number, unclear degree of blinding of raters)
[31]	PD (n = 22) vs. MSA (n = 21)	Clinical diagnose by an independent movement disorder specialist	SN + (moderate or marked)	86%, 76%, 79%; 3.63; 0.18	Moderate (no follow-up, unclear degree of blinding of raters)
	PD (n = 22) vs. MSA (n = 21)	Do	Combined finding of LN- and SN + (marked)	68%, 95%, 94%; 14.3; 0.33	Do
[27]	PD (n = 4494) vs. MSA/PSP/CBD (n = 594) (meta-analysis of 71 studies)	Clinical diagnose by an independent movement disorder specialist	SN +	75% (95% CI: 60–86%), 70% (95% CI: 55–81%)	High (systematic review and meta-analysis)
[32]	MSA/PSP (n = 92) vs. PD (n = 121)	Clinical diagnose at 4-year follow-up	Combined finding of LN + and SN- or LN + and ‚mild ‘ SN +	88%, 97%, 96%; 35.5; 0.12	Moderate (no definition of ‚mild ‘ SN +)
[33]	PD (n = 156) vs. MSA/PSP/CBD (n = 113)	Clinical diagnose at last follow-up by an independent movement disorder specialist	Combined finding of LN- and SN + (marked)	75%, 83%, 86%; 4.46; 0.30	Moderate (retrospective design, no information on the duration of follow-up)
	MSA/PSP (n = 178) vs. PD (n = 369) (pooled data of three studies)	Do	Combined finding of LN + and SN-	52%, 99%, 96%; 48.2; 0.48	Moderate (partly retrospective design, no or only short follow-up)
*PD versus dementia with Lewy bodies (DLB)*					
[34]	PD (n = 94) vs. DLB (n = 12)	Clinical diagnose by an independent movement disorder specialist	SN- or left–right asymmetric SN + (ratio ≥ 1.15)	72%, 83%, 97%; 4.34; 0.33	Low (small number of patients with DLB)
[32]	PD (n = 121) vs. DLB (n = 20)	Clinical diagnose at 4-year follow-up	SN- or asymmetric SN +	100%, 65%, 94%; 2.86; 0	Low (small number of patients with DLB, no definition of,asymmetric’ SN +)
*PD versus vascular Parkinsonism (VP)*					
[4]	PD (n = 80) vs. VP (n = 30)	Clinical diagnose by an independent movement disorder specialist	SN + (marked)	84%, 80%, 92%; 41.9; 0.20	Moderate (no follow-up)
[35]	PD (n = 78) vs. VP (n = 12)	Clinical diagnose by an independent movement disorder specialist	SN + (marked)	83%, 92%, 98%; 10.0; 0.18	Low (small number of patients with VP, unclear degree of blinding of raters)
[36]	PD (n = 372) vs. VP (n = 48) (derivation cohort)	Clinical diagnose by an independent movement disorder specialist	SN + (moderate or marked)	90%, 58%, 94%; 2.15; 0.18	Moderate (retrospective design)
	PD (n = 68) vs. VP (n = 15) (validation cohort)	Clinical diagnose at 12-months follow-up	SN + (moderate or marked)	94%, 67%, 93%; 2.82; 0.09	Low (small number of patients with VP)

3 V + denotes dilatation of third ventricle (minimum width > 10 mm); *CBD* corticobasal degeneration, *CI* confidence interval, *DLB* dementia with Lewy bodies, *LR* +  positive likelihood ratio, *LR*– negative likelihood ratiom *LN* +  hyperechogenicity of lenticular nucleus (uni- or bilateral), *LN*– normal echogenicity of lenticular nucleus (bilateral), *MSA* multiple system atrophy, *PD* Parkinson’s disease, *PPV* positive predictive value, *PS* Parkinsonian syndrome, *PSP* progressive supranuclear palsy, *SN* +  hyperechogenicity of substantia nigra (uni- or bilateral), *SN*– normal echogenicity of substantia nigra (bilateral), *TCS* transcranial sonography of brain parenchyma, *VP* vascular Parkinsonism
  Justification of recommendation: The body of evidence is largest for the use of substantia nigra TCS in the discrimination of PD versus atypical Parkinsonian syndromes; sensitivity and specificity (75%; 70%) were rated as being suboptimal in a high-quality meta-analysis [27]. The evidence on combined TCS of substantia nigra, lenticular nucleus and (optionally) third ventricle is based on a larger longitudinal study and seven cross-sectional, case–control, or smaller longitudinal studies with diverse quality of study design as well as two reviews with analysis of pooled study data. The evidence on TCS in dementia with Lewy bodies and vascular Parkinsonism is limited (altogether five studies on small cohorts). In a recent position paper of the responsible medical societies (German Society of Ultrasound in Medicine, DEGUM; German Society for Clinical Neurophysiology and Functional Imaging, DGKN) it is outlined how the status of qualified investigator for TCS in the diagnosis of PD can be achieved within the curricular educational concept of the DEGUM/DGKN (Table 2) [37].

**Table 2:** Minimum educational requirements for an investigator being regarded as ‘qualified’ for TCS in the early and differential diagnosis of PD [37]

Requirement	TCS of substantia nigra	TCS of basal ganglia
Qualification for transcranial color-coded duplex sonography certified by the DEGUM/DGKN (minimum level 1)	Mandatory	Mandatory
*AND*		
Completion of certified training courses on TCS (1 course hour = 45 min) with an at least 50% part of hands-on training in volunteers and Parkinsonian patients	≥ 8 h	≥ 16 h
*OR*^§^		
Full-time supervised individual hands-on training of several days’ duration in a clinical neurosonology lab with special expertise in TCS (as shown in scientific publications)	≥ 3 days (investigation of ≥ 8 healthy persons and ≥ 8 Parkinsonian patients)	≥ 6 days (investigation of ≥ 12 healthy persons and ≥ 20 Parkinsonian patients)
*OR*^§^		
On-the-job TCS training of several-months duration in a clinical neurosonology lab with special expertise in TCS	≥ 3 months	≥ 6 months
*AND*		
Establishment of standardized TCS in the own/local ultrasound lab	As required ^#,^*	As required *

DEGUM denotes German Society of Ultrasound in Medicine, neurology section; DGKN, German Society for Clinical Neurophysiology and Functional Imaging

^§^Proportionately interchangeable

^#^The newly qualified investigator should establish reference ranges of substantia nigra echogenic area measures in her/his ultrasound lab, using standardized system settings. For this, the substantia nigra should be assessed bilaterally in at least 50 healthy adults at various ages between 18 and 80 years (resulting in ≥ 100 measures to calculate percentiles) [13]

*The newly qualified investigator should use the possibility of consulting an expert sonographer to review and discuss unclear TCS findings (recorded in short video clips)

#### Recommendation (new in German guideline, 2023):

**Table:** 

TCS performed by a qualified investigator can be considered for supporting the differential diagnosis of PD versus atypical and secondary Parkinsonian syndromes.	
TCS for the discrimination of PD from atypical Parkinsonian syndromes shall include assessment of substantia nigra, lenticular nucleus and third ventricle.	
Level of consensus: 97.4%, strong consensus.

(2) *What is the accuracy of TCS in the differential diagnosis of PD versus essential tremor?*
  Background: The discrimination between essential tremor and early tremor-predominant PD by clinical investigation only is sometimes impossible. Therefore, there is a strong need of additional diagnostic tools to increase diagnostic certainty.
  Evidence basis: In this literature search one high-quality systematic review with meta-analysis of studies on TCS only, and three cross-sectional studies on the combination of TCS and olfactory testing, but no studies with histologically proven diagnosis were identified.
  Result: A systematic review with meta-analysis involved 18 appropriate TCS studies on 1264 patients with PD and 824 patients with essential tremor [38]. The meta-analysis found a sensitivity of 85% (95% confidence interval, 79.4–88.6%) and a specificity of 84% (78.4–88.2%) of TCS in the differentiation of PD versus essential tremor. A subgroup analysis of three out of the 18 studies showed in addition that diagnostic sensitivity and specificity are similar to that of dopamine transporter scintigraphy (DaTSCAN) [38]. For this, TCS of substantia nigra is sufficient, TCS of basal ganglia or ventricles does not add diagnostic value [39]. Diagnostic specificity is increased by combining TCS and screening for hyposmia (e.g., with Sniffin’ Sticks; Table 3) [36, 40, 41]. Patients with essential tremor and the TCS finding of SN + maybe at an increased risk of later developing PD [42]. TCS of SN is to be performed by a qualified investigator [16, 24].

**Table 3:** Studies on TCS for the differential diagnosis of PD versus essential tremor

Study	Diagnostic groups	Diagnostic gold standard	TCS criterion	Sensitivity, specificity, PPV; LR + ; LR–	Quality of study, comment
*PD versus essential tremor (ET): TCS only*					
[38]	PD (n = 1264) vs. ET (n = 824) (meta-analysis of 18 studies)	Clinical diagnose by an independent movement disorder specialist, partly DaTSCAN	SN +	85%, 84%, (86%); 4.11; 0.22	High (systematic review and meta-analysis)
*PD versus essential tremor (ET): combination of TCS and olfactory testing*					
[36]	PD (n = 372) vs. ET (n = 20) (derivation cohort)	Clinical diagnose by an independent movement disorder specialist	Combined finding of SN + (moderate or marked) and hyposmia (Sniffin’ Sticks test)	66%, 100%, 100%	Moderate (retrospective design)
	PD (n = 68) vs. ET (n = 15) (validation cohort)	Clinical diagnose at 12-months follow-up	Do	53%, 87%, 95%; 3.97; 0.54	Low (small number of patients with ET)
[40]	PD (n = 37) vs. ET (n = 26)	Clinical diagnose by an independent movement disorder specialist	Combined finding of SN + (moderate or marked) and hyposmia (Sniffin’ Sticks test)	30%, 100%, 100%	Moderate (small number of patients)
[41]	PD (n = 30) vs. ET (n = 21)	Clinical diagnose by an independent movement disorder specialist	Combined finding of SN + (marked) and hyposmia (Sniffin’ Sticks test)	63%, 100%, 100%	Low (small number of patients with ET; unclear degree of blinding of raters)

*DaTSCAN* denotes dopamine transporter scintigraphy, *ET* essential tremor, *LR* +  positive likelihood ratio, *LR*– negative likelihood ratio, *PPV* positive predictive value, *PD* Parkinson’s disease, *SN* +  hyperechogenicity of substantia nigra (uni- or bilateral), *TCS* transcranial sonography of brain parenchyma
  Justification of recommendation: The evidence on TCS of substantia nigra is based on a high-quality systematic review with meta-analysis of 18 appropriate studies. The diagnostic reliability of TCS (sensitivity, 85%, specificity, 84%) is comparable to that of dopamine transporter scintigraphy (DaTSCAN). The evidence for combining TCS and olfactory screening is based on three smaller cross-sectional and cohort studies. In a recent position paper it is outlined how the status of qualified investigator for TCS in the diagnosis of PD can be achieved within the curricular educational concept of the DEGUM/DGKN (Table 2) [37].

#### Recommendation (new in German guideline, 2023):

**Table:** 

TCS performed by a qualified investigator can be considered for supporting the differential diagnosis of PD versus essential tremor.	
TCS for the discrimination of PD from essential tremor can be combined with a screening test for hyposmia to increase diagnostic certainty.	
Level of consensus: 97.1%, strong consensus.

(3) What is the accuracy of TCS in the diagnosis of PD in persons with typical early symptoms*, compared with the diagnosis established by clinical follow-up? (*early motor signs of PD, hyposmia, depression, REM sleep behavior disorder).
  Background: The diagnosis of incident PD may allow for the early initiation of upcoming neuroprotective/neuro-restorative therapies. So far, there is no possibility of diagnosing incident PD by a single test. Therefore, there is a need of additional diagnostic tools to increase diagnostic certainty, especially in individuals with early symptoms of PD.
  Evidence basis: In this literature search one longitudinal study on a large German cohort of persons at risk of developing PD, a pooled analysis of five German longitudinal studies on risk cohorts, and three small longitudinal studies of risk cohorts, but no studies with histologically proven diagnosis were identified.
  Result: TCS shows, depending on the applied cut-off value, moderate to marked SN + at least unilaterally in 9–22% (on average, 13%) of adult healthy population, which is detected in 75–90% (on average, 83%) of patients with PD [43]. TCS of SN requires a qualified investigator [16, 24]. The finding of SN + in healthy subjects aged > 50 years indicates a 20-fold increased risk of subsequently developing PD, however the positive predictive value is low (6%) [44]. Longitudinal studies on populations with epidemiologically increased risk of subsequent PD (mild motor signs of PD, hyposmia, depression, idiopathic REM sleep behavior disorder) were found to have higher positive predictive values of SN + for indicating incident PD (Table 4) [45–49]. A pooled analysis of five German follow-up studies on risk cohorts suggests that SN + may be of higher diagnostic value in women (compared to men) and individuals at age < 65 years [48]. In cohorts with REM sleep behavior disorder proven on polysomnography, rather high positive predictive values of about 50% for incident PD or dementia with Lewy bodies have been reported [46, 49]. The predictive value for incident PD in risk cohorts can be increased by combining TCS of substantia nigra with a screening test for hyposmia (e.g. Sniffin’ Sticks) [45, 49]. Currently the combined assessment of a bunch of risk markers (including e.g. substantia nigra TCS) and prodromal markers is recommended to enhance the diagnostic certainty in an individual [50, 51]. It is to be expected that a more precise prediction of incident PD will be possible in the future through the additional inclusion of novel laboratory and genetic markers [52].

**Table 4:** Studies on TCS in comparison with clinical follow-up for the diagnosis of PD in persons with typical early symptoms of PD

Study	Study cohorts	Diagnostic gold standard	TCS criterion at baseline	Sensitivity, specificity, PPV; LR + ; LR–	Quality of study, comment
[45]	Patients with severe depression at baseline (n = 46)	Clinical diagnose of PD at 10-year follow-up (n = 3)	SN + (marked)	100%, 67%, 17%; 3,07; 0	Moderate (low number of patients with incident PD)
	Do	Do	Combined finding of SN + (marked) and hyposmia (Sniffin’ Sticks test) and mild motor signs of PD	100%, 98%, 75%; 46.0; 0	Do
[46]	Patients with iRBD confirmed on polysomnography at baseline (n = 49)	Clinical diagnose of PD or DLB at 5-year follow-up (n = 18)	SN + (moderate or marked)	44%, 68%, 44%; 1.38; 0.82	Moderate (in some cases inconstant TCS findings of SN echogenicity at repeated investigation)
[47]	Participants with TCS (n = 579), approximately 70% of these with hyposmia and/or iRBD and/or depression at baseline	Clinical diagnose of PD at 6-year follow-up (n = 9)	SN + (marked)	78%, 79%, 6%; 3.79; 0.28	High (prospective study with a relatively large sample size)
[48]	Participants with TCS (n = 2121), approximately 50% of these with mild motor symptoms of PD and/or hyposmia and/or iRBD and/or depression at baseline	Clinical diagnose of PD or DLB at 6-year follow-up (n = 30)	SN + (marked)	70%, 78%, 4%; 3.17; 0.39	High (analysis of pooled data of five prospective follow-up studies from centers with high-quality TCS)
[49]	Patients with iRBD confirmed on polysomnography at baseline (n = 34)	Clinical diagnose of PD or DLB at follow-up of up to 11 years (n = 13)	SN + (moderate or marked)	61%, 71%, 57%; 2.15; 0.54	Moderate (missing information on the applied ultrasound system)
	Do	Do	Combined finding of SN + (moderate or marked) and hyposmia (OSIT-J test)	31%, 100%, 100%	Do

*DLB* denotes dementia with Lewy bodies, *iRBD* idiopathic rapid eye movement (REM) sleep behavior disorder, *LR* +  positive likelihood ratio, *LR*– negative likelihood ratio, *OSIT-J* Olfactory Stick Identification Test for Japanese, *PPV* positive predictive value, *PD* Parkinson’s disease, *SN* +  hyperechogenicity of substantia nigra (uni- or bilateral), *TCS* transcranial sonography of brain parenchyma
  Justification of recommendation: The evidence on TCS in comparison with clinical follow-up is based on a study with pooled data analysis of five German follow-up studies in three large and two small risk cohorts, and another two small longitudinal studies on risk cohorts with an approximately 10-year follow-up. In a recent position paper it is outlined how the status of qualified investigator for TCS in the diagnosis of PD can be achieved within the curricular educational concept of the DEGUM/DGKN (Table 2) [37].

#### Recommendation (new in German guideline, 2023):

**Table:** 

TCS performed by a qualified investigator can indicate an increased individual risk of subsequent PD, however, the predictive value of stand-alone TCS is low.	
In individuals aged > 50 years with REM sleep behavior disorder proven on polysomnography, the combined findings of substantia nigra hyperechogenicity (SN +) on TCS and verified hyposmia should be regarded as indication of presence of PD.	
Level of consensus: 97.0%, strong consensus.	

## Discussion

The present updated German guideline on PD includes a newly formulated chapter on the clinical diagnostic uses of TCS (brain parenchyma sonography) in PD [21]. The analysis of scientific evidence on the three diagnostic questions was based on the results of PubMed search of records until end of 2021 and forward citation search until 30th July 2023. A repeat of the PubMed searches on 7th January 2024 yielded the same records as in the search performed during the guideline development process which makes it unlikely that a relevant recent study was missed. Compared with the previously issued versions of the German and European guidelines [17–20], the discrimination between PD and essential tremor on TCS has been addressed here for the first time in a separate section. Another new element of the present guideline is the definition of the educational requirements for being regarded as ‘qualified TCS investigator’ in PD, established by the responsible German medical societies (DEGUM, neurology section; DGKN) [37]. This definition comprises separate qualification criteria for TCS of substantia nigra and TCS of basal ganglia since the latter requires longer training. It can be expected that the present guideline recommendations will promote the diagnostic use of TCS in clinical practice.

Still, the recommendations for the diagnostic use of TCS in PD are formulated with a rather moderate degree of strength which deserves comment. There are several reasons for this. First, there are no studies so far comparing the diagnoses obtained by TCS with post-mortem histopathological investigation which, however, remains the diagnostic gold standard in Parkinsonian syndromes. For other neuroimaging methods (MRI, radionuclide scan) used in this context there are study data with post-mortem verification available [53, 54]. Even though early TCS findings have been validated by investigating asymptomatic and symptomatic gene mutation carriers in families with mono-genetically caused PD [55–58], and agreement of MRI and TCS localization of SN has been shown [59], there is a need of studies comparing TCS with post-mortem findings in PD and atypical Parkinsonian syndromes.

Second, in Germany and most European countries TCS is usually performed by neurologists, but rarely by radiologists. While transcranial color-coded duplex sonography (TCCS) of intracranial arteries is an obligatory part of residency training in neurology in Germany and many other European countries [60], TCS in movement disorders is not obligatory. Even though special courses are regularly offered by the DEGUM/DGKN and the European Society of Neurosonology and Cerebral Hemodynamics (ESNCH; https://esnch.org/), the number of physicians who fulfil the qualification criteria (Table 2) is rather limited. The now clearly defined qualification criteria for TCS in PD, along with the accompanying recommendation how the special training can be realized in the curricular concept of DEGUM/DGKN [37], may enhance the offer of such opportunities. On the other hand, there are specific advantages of TCS (compared to MRI or molecular imaging) that deserve to be made more known: TCS can be performed by the movement disorder specialists themselves, and is applicable at any location using portable ultrasound systems (e.g. in neurological practices). Moreover, the diagnostic precision of TCS is nowadays enhanced by technologies such as real-time MRI-ultrasound fusion imaging and integral digitized image analysis [61–65]. New attractive TCS applications in PD, such as time-saving postoperative control of deep brain electrode position, especially in patients with subthalamic nucleus stimulation [64, 65], may further increase interest in learning this application.

Third, characteristic findings in PD, including alteration of substantia nigra, can nowadays be visualized and quantified on MRI [53, 59, 66]. Compared with MRI, the resource of qualified TCS is less widely available, and TCS image quality may be affected by temporal skull bone thickness and experience of the investigator. Nevertheless, current evidence supports the view that elaborate MRI imaging and TCS disclose different aspects of substantia nigra pathology [67]. Since the typical TCS findings in PD (SN +) and atypical Parkinsonian disorders (LN +) are present in the early disease stages [29, 44–49, 55–58], TCS can well be employed for diagnostic screening. It should be stressed that for the planimetric measurement of substantia nigra echogenicity, which is the current standard of grading its echogenicity on TCS, high intra- and inter-rater reliability has been demonstrated with experienced investigators [16]. The potential increase of diagnostic validity of substantia nigra and basal-ganglia TCS by digitized image analysis [13, 61–63], especially if TCS is performed by less experienced investigators, remains to be proven in prospective studies. Ongoing advances in TCS technology could even reduce the impact of the main obstacle to TCS in the coming years, namely the inter-individually variable skull bone thickness [68, 69].

In conclusion, the updated guideline underpins the use of TCS in PD. The strength of recommendation of TCS in PD may potentially increase in future guideline issues. For this, TCS studies in Parkinsonian patients with subsequently autopsy-proven diagnosis as well as the adherence of potential investigators to standardized educational curricula are desired.

## Supplementary Information

Below is the link to the electronic supplementary material.

Supplementary file1 (PDF 21 KB)

Supplementary file2 (PDF 80 KB)

## Data Availability

Systematic literature search data will be shared by request from any qualified investigator. Data sharing requests are made in writing through Dr. Walter (uwe.walter@med.uni-rostock.de) and require a formal data sharing agreement with approval from the Rostock University Medical Center and the guideline office of the German Society of Neurology (DGN). Data sharing agreements must include details on how the data will be stored, who will have access to the data and intended use of the data, and agreements as to the allocation of intellectual property.
