# Supplementary material for: Systematic review-based guideline “Parkinson’s disease” of the German Society of Neurology: diagnostic use of transcranial sonography
Source: J Neurol. 2024 Jul 4;271(12):7387–401. doi: 10.1007/s00415-024-12502-1 (PMC11588812; doi:10.1007/s00415-024-12502-1)
Supplement: Supplementary file 1 — Supplementary file1 (PDF 21 KB) [file 415_2024_12502_MOESM1_ESM.pdf]

Table S1. PubMed search strategy and detected records until 31.12.2021

Keyword	Number of records
Disease-related (included)	
Parkinson disease [MeSH]	68,681
Parkinson* [tiab]	123,589
Parkinsonian disorders [MeSH]	84,142
Movement disorders [MeSH]	140,975
Disease-related (excluded)	
Wolff parkinson white syndrome [MeSH]	5,519
Wolff [tiab] AND parkinson* [tiab]	4,188
Wpw [tiab] AND syndrome [tiab]	1,406
wpw [tiab]	1,592
Hypothesis-related (included)	
Physical examination [tiab]	59,751
Clinical diagnosis [tiab]	49,189
Clinical examination [tw]	43,336
Follow-up [tiab]	1,029,501
long-term [tw]	859,869
Gold standard [tw]	71,995
Diagnosis [MeSH]	8,785,207
Diagnostic use [tiab]	2,360
Diagnostic test [MeSH] OR diagnostic test [tiab]	30,788
Differential diagnosis[MeSH] OR differential diagnosis [tiab]	517,944
Sonography [tiab]	33,588
Brain parenchyma* [tiab]	7,169
Transcranial [tiab] AND sonography [tiab]	2,553
Sonography [tiab] AND parenchyma [tiab]	574
Brain parenchyma [tiab] AND (sonography [tiab] OR tcs [tiab])	75
Transcranial B-mode sonography [tiab]	26
Brain parenchyma sonography [tiab]	13
Parkinson Disease, Secondary [MeSH]	6,572
Parkinson Disease, Familial, Type 1 [Supplementary Concept]	46
Atypical Parkinson Disease [MeSH]	883
Atypical parkinsonism [tiab]	628
Essential Tremor [MeSH] OR essential tremor [tiab]	4,419
early diagnosis [tiab]	87,699
early symptoms [tiab]	2,381
REM sleep behavior disorder [MeSH]	1,401
Hyposmia [MeSH] OR hyposmia [tiab]	1,716
Obstipation [tiab] OR constipation [tiab]	26,295
Depression [MeSH] OR depression [tiab]	430,282
Neurological examination [MeSH]	6,544

Diagnostic question 1

(((((Parkinson disease [MeSH]) OR (Parkinson* [tiab])) OR (Parkinsonian disorders [MeSH])) OR (Movement disorders [MeSH])) NOT (((((Wolff parkinson white syndrome [MeSH]) OR (wolff [tiab] AND parkinson* [tiab])) OR (wpw [tiab] AND syndrome [tiab])) OR (wpw [tiab])))) AND (((((((((((Physical examination [tiab]) OR (Clinical diagnosis [tiab])) OR (Clinical examination [tw])) OR (Follow-up [tiab])) OR (long-term [tw])) OR (Gold standard [tw])) OR (Diagnosis [MeSH])) OR (Diagnostic use [tiab])) OR (Diagnostic test [tiab] OR diagnostic test [MeSH])) OR (differential diagnosis [MeSH] OR differential diagnosis [tiab])) AND ((((brain parenchyma* [tiab]) OR (brain parenchyma [tiab] AND (sonography [tiab] OR tcs [tiab]))) OR (Transcranial B-mode sonography [tiab])) OR (Brain parenchyma sonography [tiab]))) AND ((((Parkinson Disease, Secondary [MeSH]) OR (Parkinson Disease, Familial, Type 1 [Supplementary Concept])) OR (Atypical Parkinson Disease [MeSH])) OR (Atypical parkinsonism [tiab]))) **11**

Diagnostic question 2

(((((Parkinson disease [MeSH]) OR (Parkinson* [tiab])) OR (Parkinsonian disorders [MeSH])) OR (Movement disorders [MeSH])) NOT (((((Wolff parkinson white syndrome [MeSH]) OR (wolff [tiab] AND parkinson* [tiab])) OR (wpw [tiab] AND syndrome [tiab])) OR (wpw [tiab])))) AND (((((((((((Physical examination [tiab]) OR (Clinical diagnosis [tiab])) OR (Clinical examination [tw])) OR (Follow-up [tiab])) OR (long-term [tw])) OR (Gold standard [tw])) OR (Diagnosis [MeSH])) OR (Diagnostic use [tiab])) OR (Diagnostic test [tiab] OR diagnostic test [MeSH])) OR (differential diagnosis [MeSH] OR differential diagnosis [tiab])) AND ((((brain parenchyma* [tiab]) OR (brain parenchyma [tiab] AND (sonography [tiab] OR tcs [tiab]))) OR (Transcranial B-mode sonography [tiab])) OR (Brain parenchyma sonography [tiab]))) AND ((Essential Tremor [MeSH] OR essential tremor [tiab]) OR (Tremor [MeSH] OR tremor [tiab]))) **6**

Diagnostic question 3

(((((Parkinson disease [MeSH]) OR (Parkinson* [tiab])) OR (Parkinsonian disorders [MeSH])) OR (Movement disorders [MeSH])) NOT (((((Wolff parkinson white syndrome [MeSH]) OR (wolff [tiab] AND parkinson* [tiab])) OR (wpw [tiab] AND syndrome [tiab])) OR (wpw [tiab])))) AND ((((((((Physical examination [tiab]) OR (Clinical diagnosis [tiab])) OR (Clinical examination [tw])) OR (Follow-up [tiab])) OR (long-term [tw])) OR (Gold standard [tw])) AND (((((((early diagnosis [tiab]) OR (early symptoms [tiab])) OR (REM sleep behavior disorder [MeSH])) OR (hyposmia [MeSH] OR hyposmia [tiab])) OR (obstipation [tiab] OR constipation [tiab])) OR (depression [MeSH] OR depression [tiab])) OR (Neurological examination [MeSH]))) AND (((((((brain parenchyma [tiab] AND (sonography [tiab] OR tcs [tiab])) OR (transcranial [tiab] AND sonography [tiab])) OR (sonography [tiab] AND parenchyma [tiab])) OR (sonography [tiab])) OR (brain parenchyma* [tiab])) OR (Transcranial B-mode sonography [tiab])) OR (Brain parenchyma sonography [tiab]))) **30**

MeSH denotes Medical Subject Headings; tiab, title or abstract containing the given keyword; tw, text containing the given keyword.

Bold values: number of records identified on PubMed search which were further screened (see Figure S1, Online Resource 2)
