# Supplementary material for: Systematic review-based guideline “Parkinson’s disease” of the German Society of Neurology: diagnostic use of transcranial sonography
Source: J Neurol. 2024 Jul 4;271(12):7387–401. doi: 10.1007/s00415-024-12502-1 (PMC11588812; doi:10.1007/s00415-024-12502-1)

Figure S1. PRISMA diagrams for the 3 diagnostic questions (reporting template obtained from [23])

A) Diagnostic question 1: What is the accuracy of TCS in the differential diagnosis of PD versus atypical and secondary Parkinsonian syndromes?

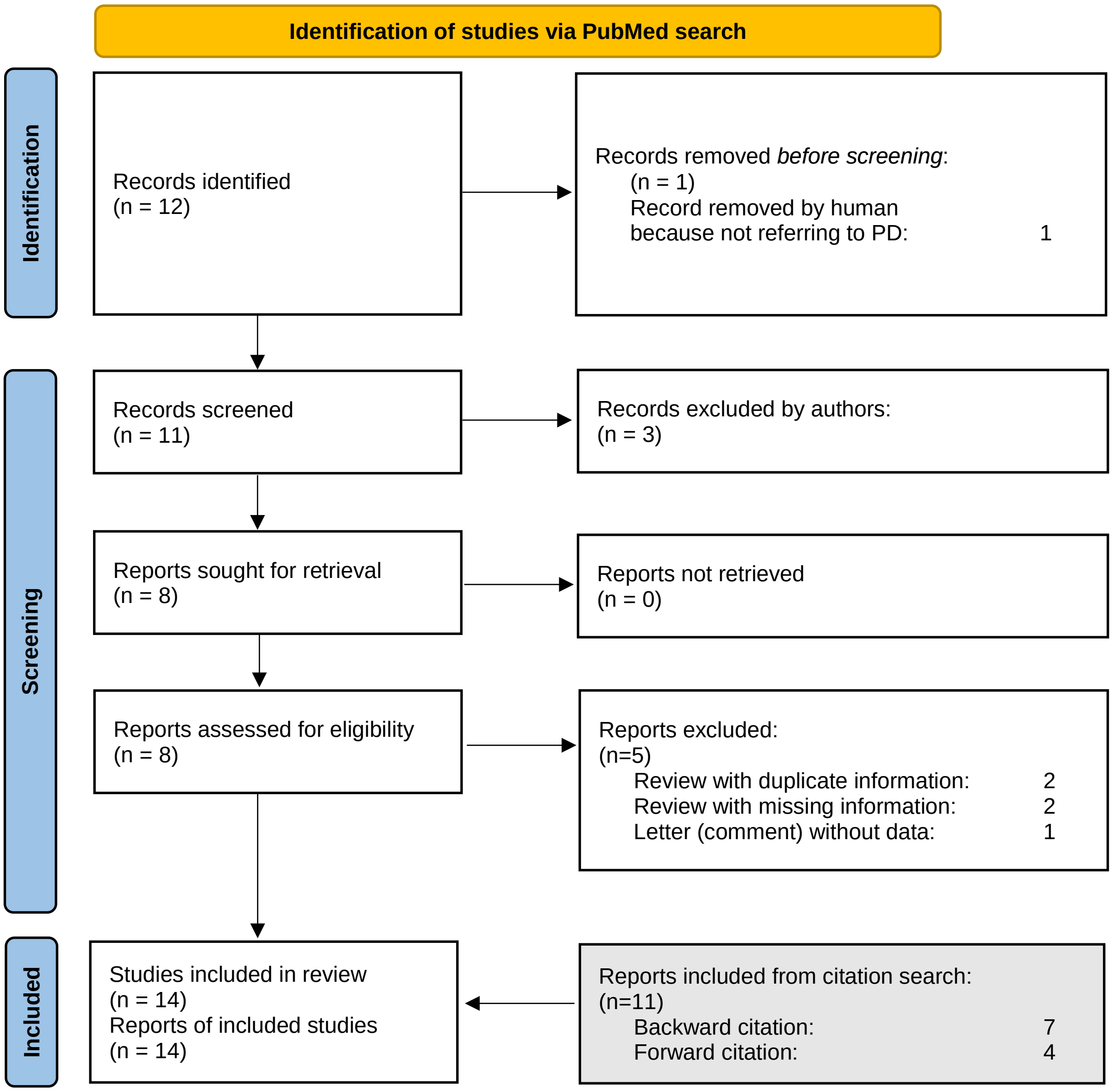

B) Diagnostic question 2: What is the accuracy of TCS in the differential diagnosis of PD versus essential tremor?

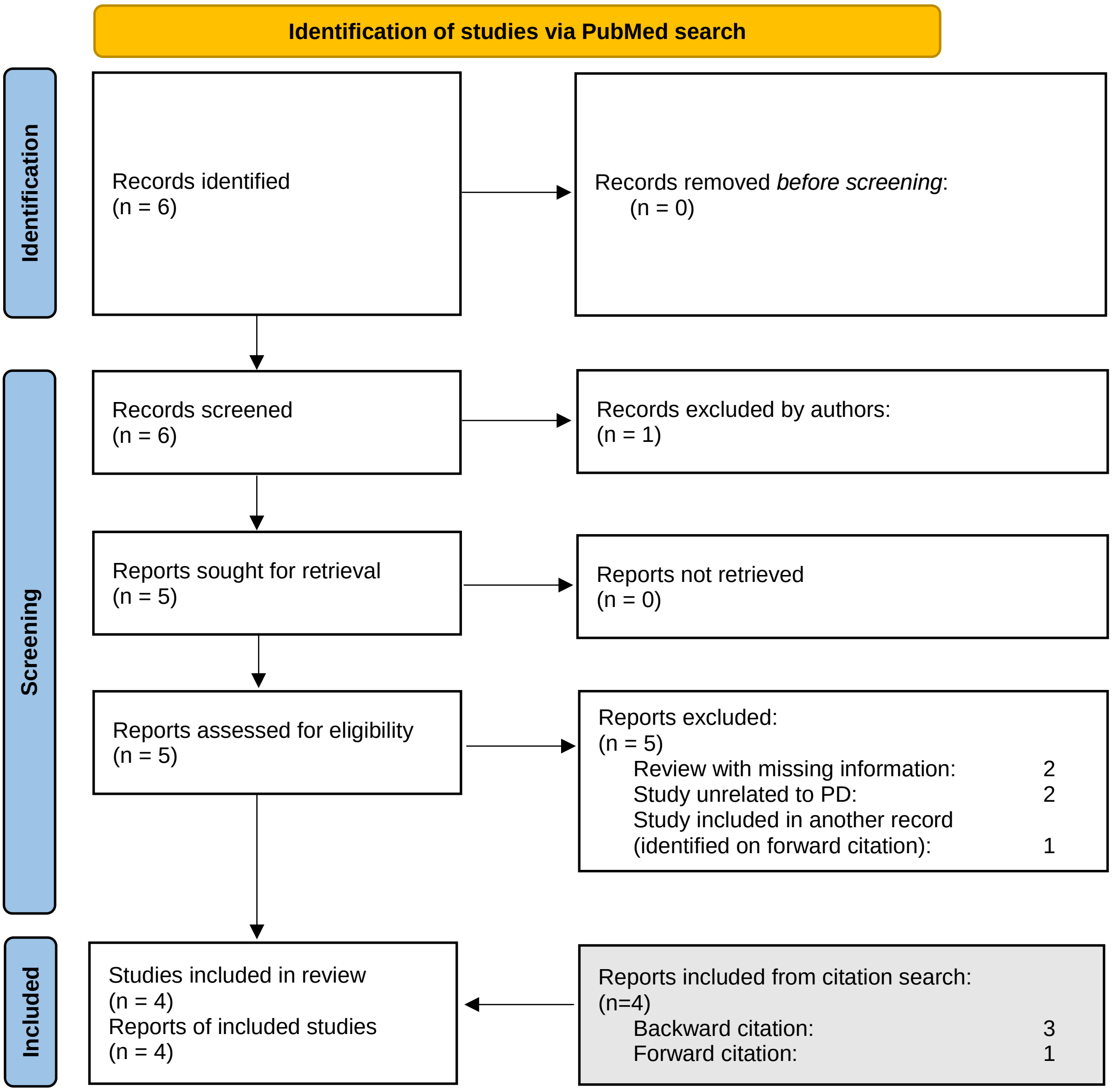

C) Diagnostic question 3: What is the accuracy of TCS in the diagnosis of PD in persons with typical early symptoms*, compared with the diagnosis established by clinical follow-up?
(* early motor signs of PD, hyposmia, depression, REM sleep behavior disorder)

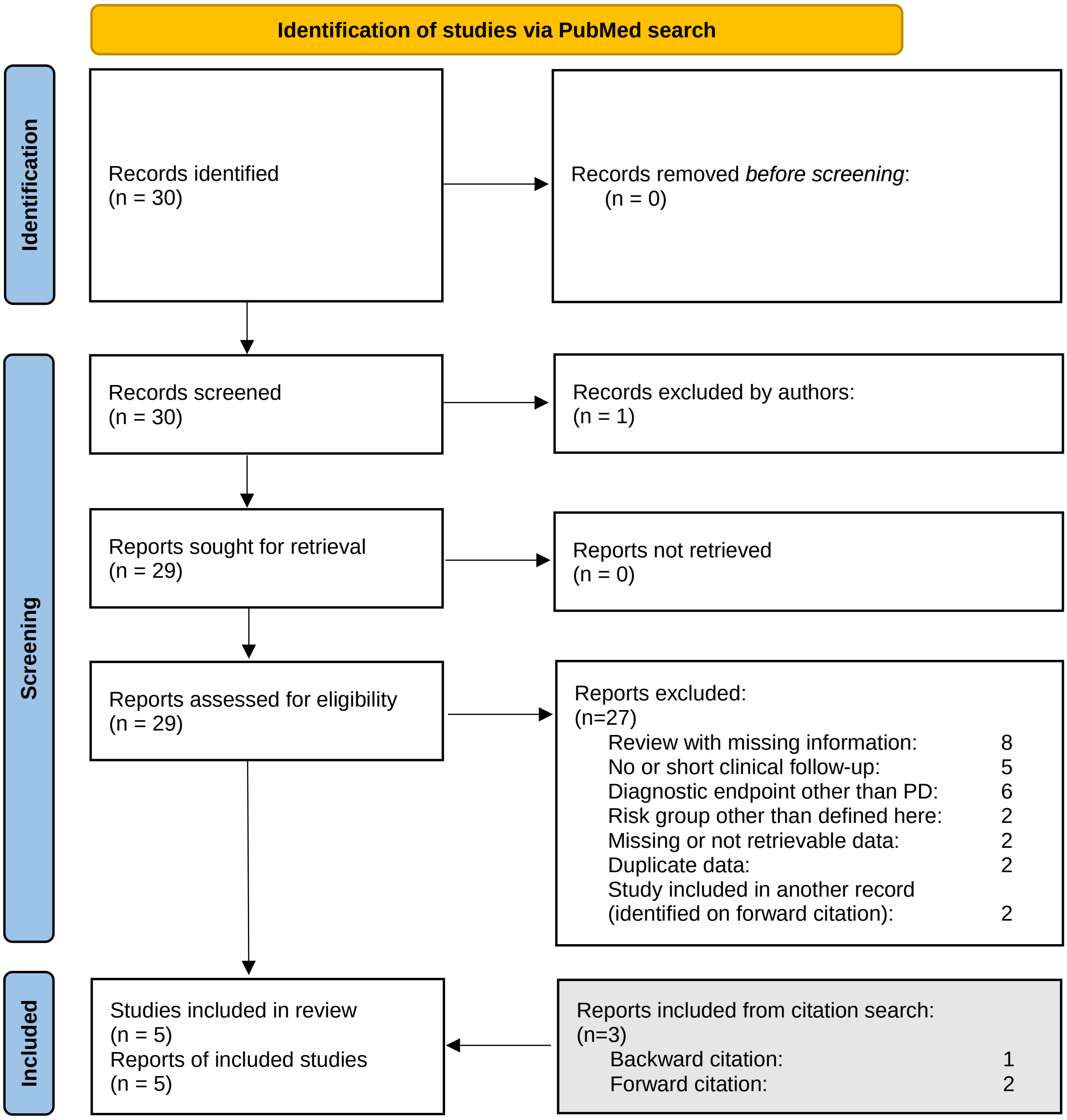

Supplement: Supplementary file 2 — Supplementary file2 (PDF 80 KB) [file 415_2024_12502_MOESM2_ESM.pdf]
